# Using eye tracking to test for individual differences in attention to attractive faces

**DOI:** 10.3389/fpsyg.2015.00042

**Published:** 2015-02-02

**Authors:** Christian Valuch, Lena S. Pflüger, Bernard Wallner, Bruno Laeng, Ulrich Ansorge

**Affiliations:** ^1^Cognitive Science Research Platform, University of ViennaVienna, Austria; ^2^Department of Basic Psychological Research and Research Methods, Faculty of Psychology, University of ViennaVienna, Austria; ^3^Department of Anthropology, Faculty of Life Sciences, University of ViennaVienna, Austria; ^4^Department of Behavioural Biology, Faculty of Life Sciences, University of ViennaVienna, Austria; ^5^Department of Psychology, Faculty of Social Sciences, University of OsloOslo, Norway

**Keywords:** attention, faces, gender, eye color, attractiveness, gap effect, dot probe, linear mixed effects models

## Abstract

We assessed individual differences in visual attention toward faces in relation to their attractiveness via saccadic reaction times. Motivated by the aim to understand individual differences in attention to faces, we tested three hypotheses: (a) Attractive faces hold or capture attention more effectively than less attractive faces; (b) men show a stronger bias toward attractive opposite-sex faces than women; and (c) blue-eyed men show a stronger bias toward blue-eyed than brown-eyed feminine faces. The latter test was included because prior research suggested a high effect size. Our data supported hypotheses (a) and (b) but not (c). By conducting separate tests for disengagement of attention and attention capture, we found that individual differences exist at distinct stages of attentional processing but these differences are of varying robustness and importance. In our conclusion, we also advocate the use of linear mixed effects models as the most appropriate statistical approach for studying inter-individual differences in visual attention with naturalistic stimuli.

## INTRODUCTION

At all times, humans are capable of processing only a limited amount of their visual environment. This selectivity is called visual attention and because of its widespread involvement in cognitive tasks, ranging from reading and communication to scene perception and navigation, experimental psychology has aimed to understand the principles governing attention. Although many models of visual attention have been advocated, many open questions remain. One open question concerns the origins of inter-individual differences in visual attention. Here, we tested whether principles suggested by evolutionary psychology explain some of the inter-individual differences in attention.

Previous research has demonstrated that human faces are among the most interesting stimuli for visual attention (e.g., Bindemann et al., 2005; Ro et al., 2007; Langton et al., 2008; Thoma and Lavie, 2013). However, the degree to which a particular face receives attention might differ between individuals. First, evolutionary psychology suggests that certain phenotypical features in faces are perceived as particularly attractive (because they signal health or reproductive quality; Fink and Penton-Voak, 2002; Rhodes, 2006) but the relevance of such visual cues differs between individuals according to their sexes (Buss, 2003). For example, one study has shown that blue-eyed males find blue-eyed females particularly attractive and are more likely to choose them as partners while a comparably specific preference was not observed with blue- or brown-eyed women, or brown-eyed men (Laeng et al., 2007). According to an evolutionary explanation, this could be due to the recessive inheritance of genes for blue-eyes: when both partners have blue eyes, the common offspring will have blue eyes as well and this might serve as an additional assurance of paternity for the blue-eyed male. Second, research has demonstrated that humans spend more time looking at faces that are considered attractive than at less attractive faces (e.g., Aharon et al., 2001; Shimojo et al., 2003; Sui and Liu, 2009; Leder et al., 2010; Chen et al., 2012). However, these effects are not the same in all individuals: compared to women, men exhibit a higher motivation to view attractive opposite-sex faces (Levy et al., 2008; Hahn et al., 2013) and are more likely to show attentional biases toward attractive opposite-sex stimuli (Maner et al., 2003, 2007).

The two observations mentioned above were the point of departure for our study. We aimed at addressing the following open questions in this area. First, it is not known which kind of attentional sub-process differs between individuals when they are confronted with faces of varying attractiveness. For example, Leder et al. (2010) have embedded highly and less attractive faces in photographs of natural scenes and reported increased looking times at the attractive faces. Similarly, Maner et al. (2003) recorded participants’ eye movements while looking at displays containing four highly attractive and four less attractive faces that were either male or female. They found that male participants looked significantly longer at attractive female faces than at less attractive female faces. This bias was not found with male faces. In contrast, women looked longer at highly attractive female as well as male faces. However, none of the published studies provides conclusive evidence whether these results are due to more robust capture of attention by the highly attractive faces, or to the stronger holding of attention once it has been captured. Hence, in the present study we disentangle these processes by directly measuring the participants’ ability to disengage attention from attractive versus less attractive faces in Experiment 1, as well as the relative potential of different classes of faces for attentional capture in Experiment 2. Second, previous research has remained inconclusive about whether individual preferences for particular face features, as measured by attractiveness ratings, result in equivalent inter-individual differences in attention measures. Hence, we compared our measures of disengagement and capture of attention to attractiveness ratings collected from the same participants. We focused our investigation on the variables of gender and eye color because prior research suggested a high statistical power of these effects. To that end, we tested whether interactions between participant’s sex and eye color and the respective facial characteristics co-determine any of the two attentional sub-processes in response to faces.

In the current study, we also aimed to establish new statistical benchmarks for conclusions on attentional processes in response to faces. Our review of the literature showed that all prior studies in this area based their conclusions on conventional statistics. However, these methods are not optimally suited for studying inter-individual attentional differences as a function of naturalistic stimuli, such as faces. Faces vary on a variety of unknown characteristics ranging from low-level features, such as lightness, and feature combinations, such as lip versus eye curvature, to holistic characteristics (e.g., the overall facial silhouette, the eyes’ distance, and the proportional size of the nose). Because some of these features might not even be known, it is almost impossible to control for all of them. However, one statistical approach is particularly suited for studying inter-individual differences with such less controlled stimuli, namely linear mixed effects models (LMMs). This approach allows incorporating the variance explained by particular stimuli (here: faces) into a model (here: of predicting looking behavior through participant sex and/or eye color; cf. Baayen et al., 2008; Baayen and Milin, 2010; Kliegl et al., 2010). Our study should therefore also be regarded as an appeal for the use of LMM approaches when naturalistic stimuli are used to test for individual differences in attentional processing.

## EXPERIMENT 1: DISENGAGEMENT OF ATTENTION FROM FACES

To collect a direct measure of attentional disengagement, we employed a *gap saccade task* (Saslow, 1967) in which participants are instructed to make a saccade away from a centrally fixated stimulus to a second stimulus that appears in the visual periphery. The main manipulation concerns the centrally fixated stimulus, which is either extinguished (typically) 200 ms prior to the onset of the peripheral stimulus (in ‘gap’ trials), or remains visible until after the onset of the peripheral stimulus (in ‘overlap’ trials). Here, the often-replicated gap effect consists in saccades to peripheral stimuli having shorter latencies or saccadic reaction times (SRTs) in gap than overlap trials. Although the effect depends on properties of the oculomotor system (Dorris and Munoz, 1995; Walker et al., 1995) recent evidence corroborated a causal role of attention in the gap effect (Pratt et al., 2006; Jin and Reeves, 2009).

We adapted the gap-saccade task to directly measure the influence of (a) overall facial attractiveness, (b) face gender, and (c) face eye color on disengagement of attention from a centrally presented face image. Our participants were required to disengage their attention from a fixated face and make a saccade to an abruptly appearing peripheral dot target. Consistent with the classical task, the face was either switched off 200 ms prior to the onset of the peripheral target (in gap trials) or remained visible until after the onset of the peripheral target (in overlap trials). In addition to the gap effect we predicted that attractive faces would delay disengagement more effectively than less attractive faces, resulting in higher SRTs. Motivated by the research outlined in the Introduction, we also tested for an interaction between participant sex and face gender, henceforth referred to as gender interaction (GI) as well as the more complex interaction between participant sex and eye color and face gender and eye color, henceforth named eye color and gender interaction (EGI).

### METHODS

#### Participants

Forty participants with a mean age of 24 years (*SD* = 3.7) were assigned to four groups of ten that resulted from crossing the variables participant eye color (blue/brown) and sex (male/female). We chose a group sample size of ten based on a previous study (Laeng et al., 2007) which reported an effect size of Cohen’s *d* = 1.11 for the critical difference in the group of blue-eyed men using a sample size of 22 participants in a rating study. Assuming an effect of the same size in the present population, a group sample size of 10 would imply a statistical power of (1 – β) = 0.84 to reveal this difference in a two-tailed test (for β/α = 1 in a compromise analysis as implemented in Faul et al., 2007). Here and in Experiment 2, participants were undergraduate Psychology students, recruited at University of Vienna that participated voluntarily (in exchange for partial course credit). Upon arrival in the lab, they were inspected for their eye color. Participants with eye colors not clearly recognizable as blue or brown were assigned to a different experiment unrelated to the present study. All participants had normal or corrected-to-normal visual acuity and intact color vision. All participants were Caucasian and naïve with respect to the research hypotheses. The experiment was conducted in accordance with the Declaration of Helsinki and APA ethical standards in the conduct of research. Written informed consent was obtained from all participants.

#### Face stimuli

Two different sets (‘natural faces’ vs. ‘morphed faces’) of feminine and masculine faces with blue or brown eyes served as stimuli. Each set comprised 24 face images with six different faces per eye color and gender combination. Both sets were derived from the same source photographs. The source photographs were full frontal face portraits of male and female Caucasian adults aged 20–30 years with neutral expressions. For clarity, we henceforth refer with the words ‘male,’ ‘female,’ and ‘sex’ to our study participants and with the words ‘masculine,’ ‘feminine,’ and ‘gender’ to the face images. Photographs were taken under constant lighting conditions, exposure settings, shooting distance, and viewing angle. The digital camera (Canon EOS 550D with a Canon 50 mm f/1.8 lens) was calibrated using a ColorChecker Passport (X-Rite Inc., Grand Rapids, MI, USA) color standard. Exposure settings were set to f/20, 1/20 s, ISO 100 and lighting was delivered by two Bowens GM500 Digital (Bowens International Ltd., Essex, UK) flashes with softboxes mounted on tripods. The set of morphed faces was created using FantaMorph 5.0 (Abrosoft Co., Beijing, China) by averaging three different (either only masculine or only feminine) source photographs to one masculine or feminine face. None of the source photographs was used in more than one morphed face.

All natural and morphed faces used for the present study were selected from a larger pool of candidate stimuli that underwent pretests for attractiveness ratings on a 7-point scale (1 = ‘very unattractive’; 7 = ‘very attractive’) by an independent sample of participants from the same undergraduate student population (*n* = 60 for natural; *n* = 24 for morphed faces). In line with previous literature (e.g., Langlois and Roggman, 1990), average rated attractiveness of morphed faces (*M* = 4.07, *SD* = 0.78) was significantly higher than of natural faces [*M* = 3.57, *SD* = 0.94, *t*(135.7) = 3.47, *p* < 0.001]. Because of a higher variance in attractiveness judgments in natural faces than in morphed faces, the final set of natural faces comprised a broader variation in (pre-rated) attractiveness but approximately equal numbers of attractive and less attractive faces.

**FIGURE 1:**
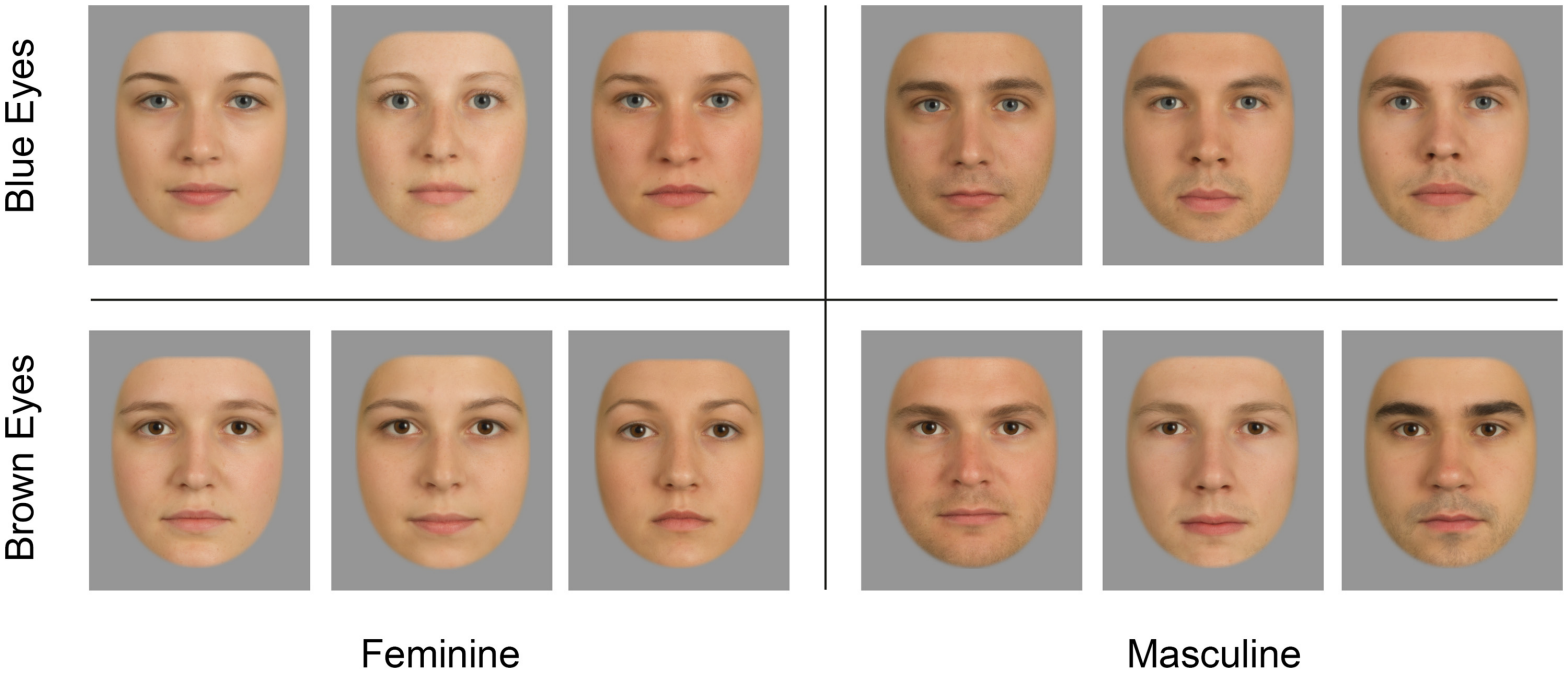
**Morphed faces served as stimuli in Experiments 1 and 2.** Each face was a composite produced by averaging three natural faces using image processing software. There were two versions of each face, one with blue eyes and one with brown eyes (only one version is depicted). Natural faces (which were the basis for the morphs but also served as a separate set of stimuli in Experiment 1) are not depicted due to privacy reasons.

Each face stimulus was presented in two variations (which differed only by eye color) to the same participants. All face stimuli were processed in Adobe Camera RAW and Adobe Photoshop CS5 (Adobe, Inc.) to standardize their appearance and to exclude possibly confounded influences on perceived attractiveness and/or attention. For that, a standardized mask was placed on hair, clothes, and background regions of the images and they were presented on a 50% gray background in the experiment (see **Figure 1**). The irises within all faces were retouched by inserting a standardized blue or brown iris with constant pupil size and iris color.

#### Apparatus

The experiment was programmed in MATLAB (MathWorks, Natick, MA, USA) using the Psychophysics Toolbox (Brainard, 1997; Pelli, 1997) and the Eyelink toolbox (Cornelissen et al., 2002) running on a personal computer under Windows XP (Microsoft, Inc.). Stimuli were displayed on a 19-in. color CRT monitor (Sony Multiscan G400) with the screen resolution set to 1,280 × 1,024 pixels at a vertical refresh rate of 85 Hz and 32 bit color depth. The monitor was calibrated using an i1Pro spectrophotometer (X-Rite Inc., Grand Rapids, MI, USA). Viewing distance was held constant at 64 cm with chin and forehead rests. Gaze data were recorded monocularly using an EyeLink 1000 Desktop Mount (SR Research Ltd., Kanata, ON, Canada) video-based eye tracker sampling at 1000 Hz. Prior to the start of the experiment, the system was calibrated on the participants’ dominant eye using a standard 5-point calibration sequence. In the course of the experiment, a manual drift check was conducted prior to every 12th trial. If the recorded gaze position differed by more than 1° from a central fixation target, recalibrations were performed (other trials started automatically after the participant had fixated on the central target for 1.5 s).

#### Experimental procedure

**FIGURE 2:**
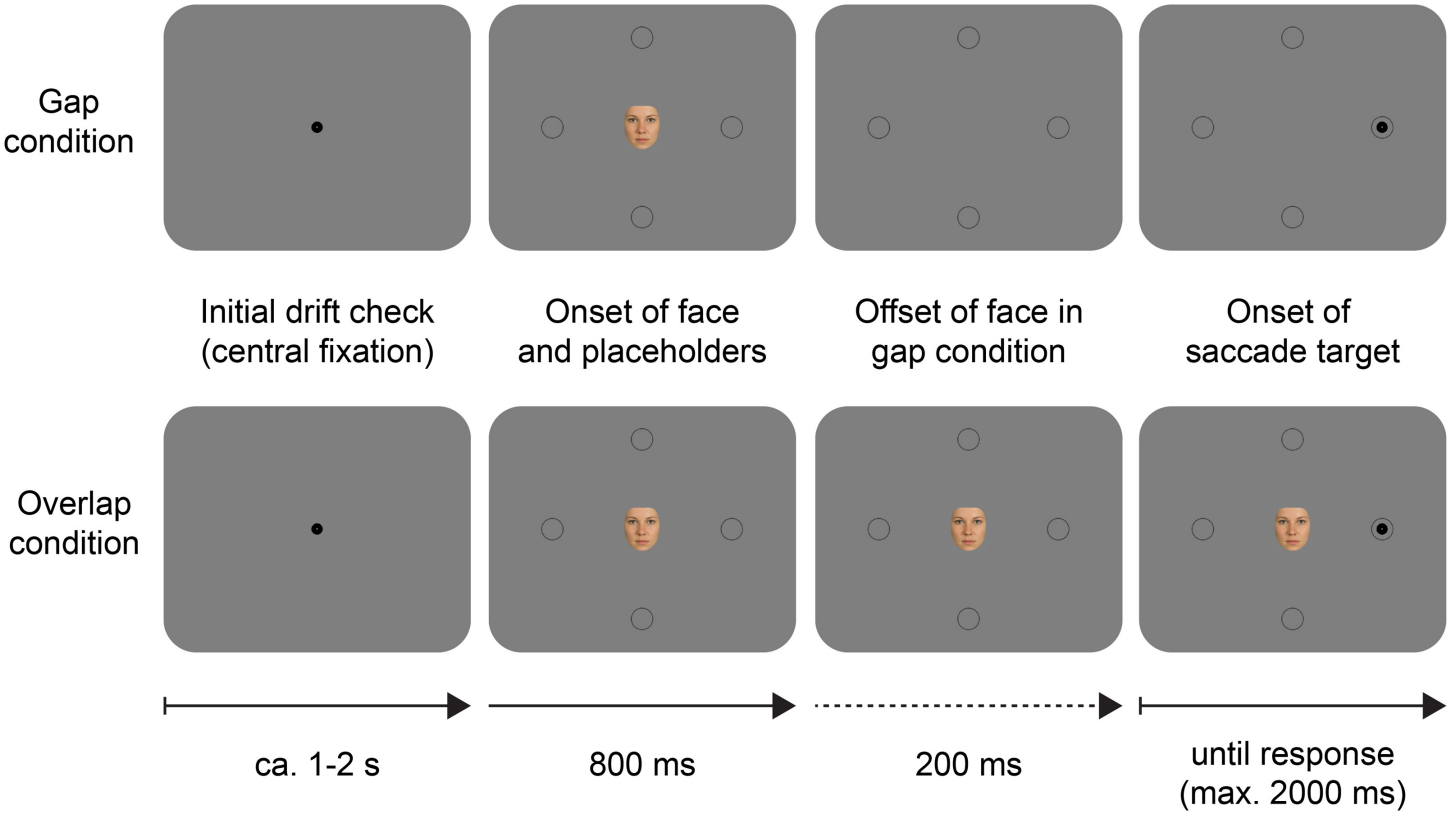
**Example schematic trials in the gap saccade task used in Experiment 1.** The saccade target appeared randomly in one of the four placeholders around the central face stimulus. In the gap condition, the face disappeared 200 ms prior to the onset of the saccade target whereas in the overlap condition the face remained on screen until the end of the trial. Arrows indicate the flow of time.

Participants were informed that the purpose of the experiment was to study the effect of human faces on visual attention and were given basic task instructions (i.e., ‘fixate on the face until a dot appears; as soon as you see a dot, look at it as quickly as possible’). They were not informed about the specific hypotheses and experimental manipulations. The experiment comprised 384 trials in randomized order which were presented in four blocks of 96 trials (between blocks, participants were allowed to rest briefly). Morphed and natural faces were presented randomly intermixed across all trials. The sequence within a trial is depicted in **Figure 2**. Each trial started with a central fixation (or drift check). Next, a face image (2.7 × 3.2°) was presented at screen center together with four equidistant dark gray circular placeholders marking the possible target locations. Placeholders had diameters of 1.7°, line strengths of 0.1°, and were placed above, below, left, and right, all at center-to-center distances of 6.8° from the face. In every trial, a black circular target dot with a diameter of 0.85° appeared 1 s after the onset of the face, randomly in one of the four placeholders. Participants executed a saccade away from the face to the peripheral target and the trial ended automatically once a target fixation was registered (fixations were detected online in an invisible square region of 2.5 × 2.5° around the target). At the start of each experimental session, participants completed 16 training trials. Training trials were identical to experimental trials except that participants were given positive feedback, whenever their saccade was correct (‘okay,’ displayed at screen center) and negative feedback if they aborted fixation prior to target onset (‘too fast’) or if no target fixation was registered within 2 s after target onset (‘timeout’). In the experimental blocks only negative feedback was provided, and these trials were automatically repeated at the end of the block.

#### Rating procedure

After completing the four experimental blocks, participants were presented a final rating block, in which each face was shown one by one in randomized order at screen center (just as in the experimental procedure) together with a 7-point rating scale (ranging from 1 = ‘very unattractive’ to 7 = ‘very attractive’). Participants judged the attractiveness of each face by pressing the according number button on a standard PC keyboard. The rating task here (and in Experiment 2) was self-paced and participants could freely choose how long they wanted to view each face before giving their rating. In total, a complete run (including setup, experiment, rating, and participant debriefing) lasted about 70 min.

#### Data analysis

Raw gaze data was parsed into sequences of saccades and fixations using the SR Research algorithm (SR Research Ltd., Kanata, ON, Canada). Saccades were determined by criteria of change in gaze position (>0.1°), eye velocity (>30°/s), and acceleration (>8,000°/s^2^). Gaze data were pre-processed in MATLAB. We analyzed SRTs, defined as the difference between the onset time of the saccade target and the onset time of the first saccade that landed on the target. In total, we recorded SRTs from 15,360 trials (384 trials from each of the 40 participants). Out of these, 557 trials (3.6%) were excluded because SRT was faster than 50 ms (most likely due to anticipations or measurement artifacts or because of blinks ahead of the saccade or during it). SRTs and ratings were analyzed in *R version 3.1.1* (R Core Team, 2014) using the *lme4 package version 1.1-7* (Bates et al., 2014) for fitting and analyzing LMMs. We applied the Satterthwaite (1946) approximation for computing *p*-values for *t*-statistics and the Kenward and Roger (1997) approximation for *F*-statistics, as implemented in the *lmerTest package version 2.0-6* (Kuznetsova et al., 2014). Preliminary analyses of SRTs and inspections of Q–Q plots and histograms revealed that a Log-transformation (natural logarithm) of SRTs was necessary to approximate a normal distribution and to achieve normally distributed model residuals. This is a common transformation for distributions of reaction times (RTs) which are often positively skewed (Baayen and Milin, 2010). In the analysis of SRTs and ratings, we included random intercepts for subjects and stimuli (individual face images).

### RESULTS

For each of the two datasets (‘natural faces’ vs. ‘morphed faces’) we selected an appropriate model using the Akaike information criterion (AIC; Akaike, 1974; Stephens et al., 2005) and significance tests based on the χ^2^-distributed likelihood-ratio between two models (cf. Glover and Dixon, 2004; Baayen and Milin, 2010). For all datasets, the general approach was the same: first, we defined a null model, which included only the random effects (as well as a fixed effect for the gap manipulation, for the SRT data). Then, we defined a sequence of nested models by step-wise increasing the complexity of the fixed effects structure. Interactions between phenotypic features of the face stimuli and the participants’ traits were coded within single variables (i.e., a four-step variable GI for the four possible combinations of participant sex and face gender; and EGI for the 16 possible combinations of participant sex and eye color and face gender and eye color). We ordered these variables and applied a successive differences contrast coding scheme. Each more complex model was a special case of the previous, simpler model. After each step, we checked whether the change in model fit justifies a decision in favor of the more complex model instead of the simpler model. The fixed effects of the best model were subsequently analyzed in more detail.

#### Subjective attractiveness ratings

**FIGURE 3:**
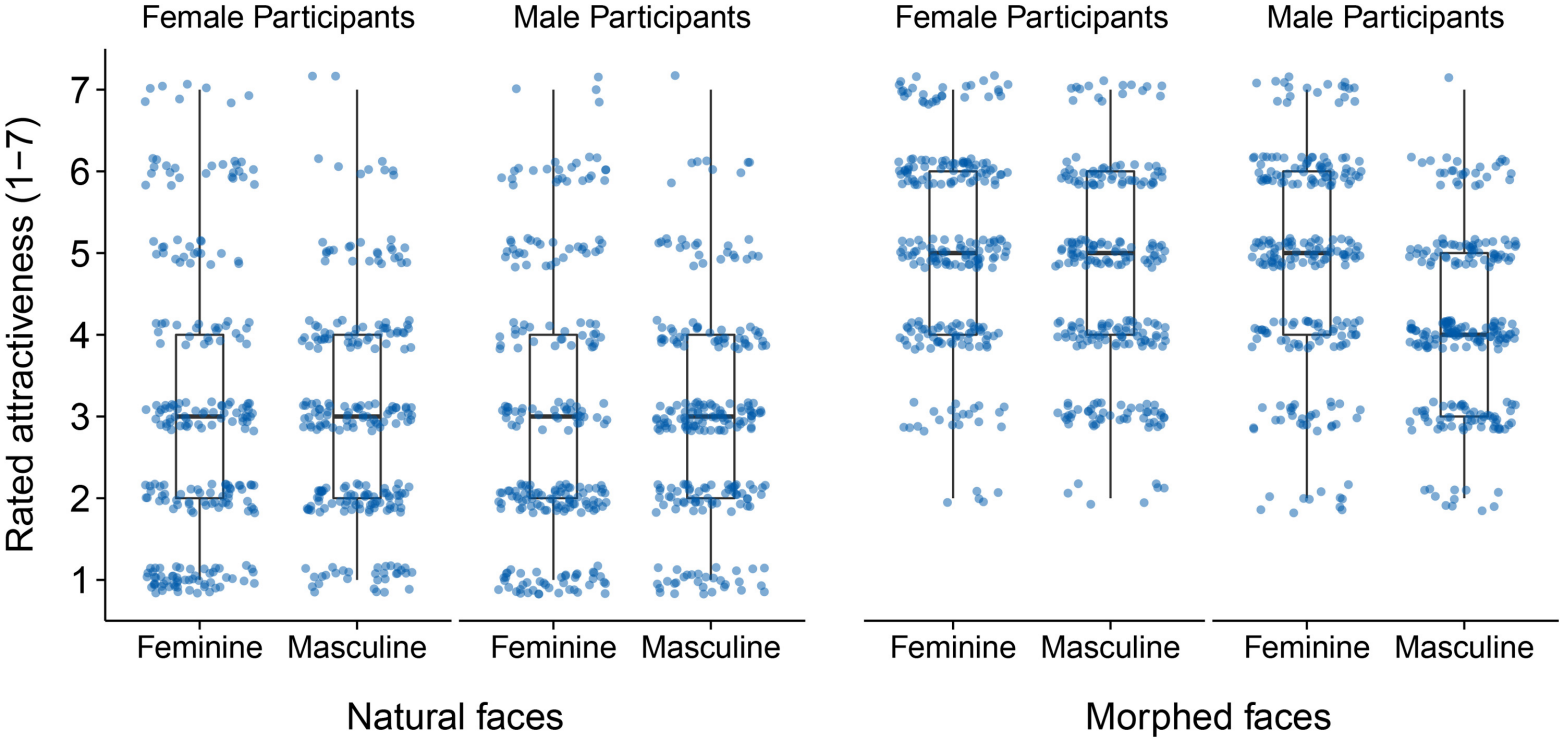
**Distribution of all subjective attractiveness judgments collected in Experiment 1.** Dots represent raw data points (*n* = 960 in each of the two stimulus sets) and are displayed jittered on the discrete rating scale for better visualization.

***Model selection: evidence for gender interaction in morphed faces.*** Results are presented in **Table 1**. For natural faces the data yielded no evidence for any of the interaction effects (as evident by the non-significant χ^2^-statistic in the likelihood-ratio test and the increasing AIC). However, for morphed faces the data suggested an interaction between participant sex and face gender (reflected in the decreasing AIC and the significant χ^2^-statistic in the likelihood-ratio test). Noteworthy, there was no evidence for the more complex gender and eye color interaction in this data either. Hence, we concluded that (at least for the highly attractive morphed faces) a model including a gender-based interaction explained subjective attractiveness ratings best (cf. **Figure 3**). For this model, we examined the gender-based individual differences in attractiveness ratings more closely.

**Table 1:** Comparison of nested linear mixed effects models (LMMs) fitted to subjective attractiveness ratings in Experiment 1.

			Model comparison			
Model	*df*	Formula	AIC	χ^2^	*df*	*p*
**Natural faces**
nat_ 0_	4	Attractiveness ∼ 1 + RE	2956			
nat_ 1_	7	Attractiveness ∼ 1 + GI + RE	2962	0.05	3	0.997
nat_ 2_	19	Attractiveness ∼ 1 + EGI + RE	2979	6.54	12	0.886
**Morphed faces**
mor_0_	4	Attractiveness ∼ 1 + RE	2756			
mor_1_	7	Attractiveness ∼ 1 + GI + RE	2748	14.1	3	0.003
mor_2_	19	Attractiveness ∼ 1 + EGI + RE	2755	17.5	12	0.133

*Attractiveness, individual subjective attractiveness judgments; Formula, model definition for the *lme4* software package; RE, random effects structure [(1| Participant) + (1| Stimulus)]; GI = [Participant Sex × Face Gender]; EGI = [Participant Sex × Participant Eye Color × Face Gender × Face Eye Color].*

***Model results: stronger preference for highly attractive feminine faces in men.*** Tests of the fixed effects part of the model mor_1_ confirmed the significant interaction between participant sex and face gender, *F*(3,67.1) = 4.82, *p* = 0.004. Model coefficients suggested that feminine faces were rated as more attractive than masculine faces by male as well as female participants. However, this bias toward feminine faces was stronger in male than in female participants (see **Table 2**).

**Table 2:** Fixed effect estimates for a rating bias toward feminine morphed faces in Experiment 1.

Fixed effect	*B (SE)*	*t*	*p*
(Intercept)	4.67 (0.144)		
Female participants (feminine – masculine)	0.47 (0.219)	2.15	0.041
Male participants (feminine – masculine)	0.72 (0.219)	3.29	0.003

*Results based on individual ratings on a 7-point scale. Masculine, ratings of masculine morphed faces. Feminine, ratings of feminine morphed faces. Positive *B* and *t* values indicate that feminine faces received higher ratings than masculine faces.*

#### Saccadic reaction times

***Model selection: facial attractiveness affects saccadic reaction times.*** Log-transformed SRTs (logSRTs) were analyzed separately for natural faces and morphed faces. Baseline models included the fixed effect of the gap manipulation, random intercepts for participants and stimuli as well as a random slope for the gap manipulation. Due to the generally larger variation in attractiveness judgments for the natural faces we included overall facial attractiveness (as a two-step variable, ‘attractive’ vs. ‘unattractive’). This categorization was based on *z*-transformed ratings given by each participant at the end of the experiment and computing a mean attractiveness score for each face. Faces with mean attractiveness scores above the median of all values were assigned to the ‘attractive’ group while the other faces were assigned to the ‘unattractive’ group.

Results are presented in **Table 3**. For natural faces, the data yielded evidence for an effect of overall facial attractiveness. Apart from that, there were no indications for gender-based (or eye color and gender based) interactions in either of the datasets.

**Table 3:** Comparison of nested LMMs fitted to log-transformed SRTs in Experiment 1.

			Model comparison			
Model	*df*	Formula	AIC		*df*	*p*
**Natural faces**
nat_ 0_	7	logSRT ∼ 1 + Gap + RE	3315			
nat_1_	9	logSRT ∼ 1 + Gap × Attractiveness + RE	3311	7.40	2	0.025
nat_2_	21	logSRT ∼ 1 + Gap × Attractiveness × GI + RE	3324	11.68	12	0.472
nat_3_	69	logSRT ∼ 1 + Gap × Attractiveness × EGI + RE	3368	52.06	48	0.319
**Morphed faces**
mor_0_	7	logSRT ∼ 1 + Gap + RE	3679			
mor_1_	13	logSRT ∼ 1 + Gap × GI + RE	3687	4.34	6	0.631
mor_2_	37	logSRT ∼ 1 + Gap × EGI + RE	3714	20.75	24	0.652

**FIGURE 4:**
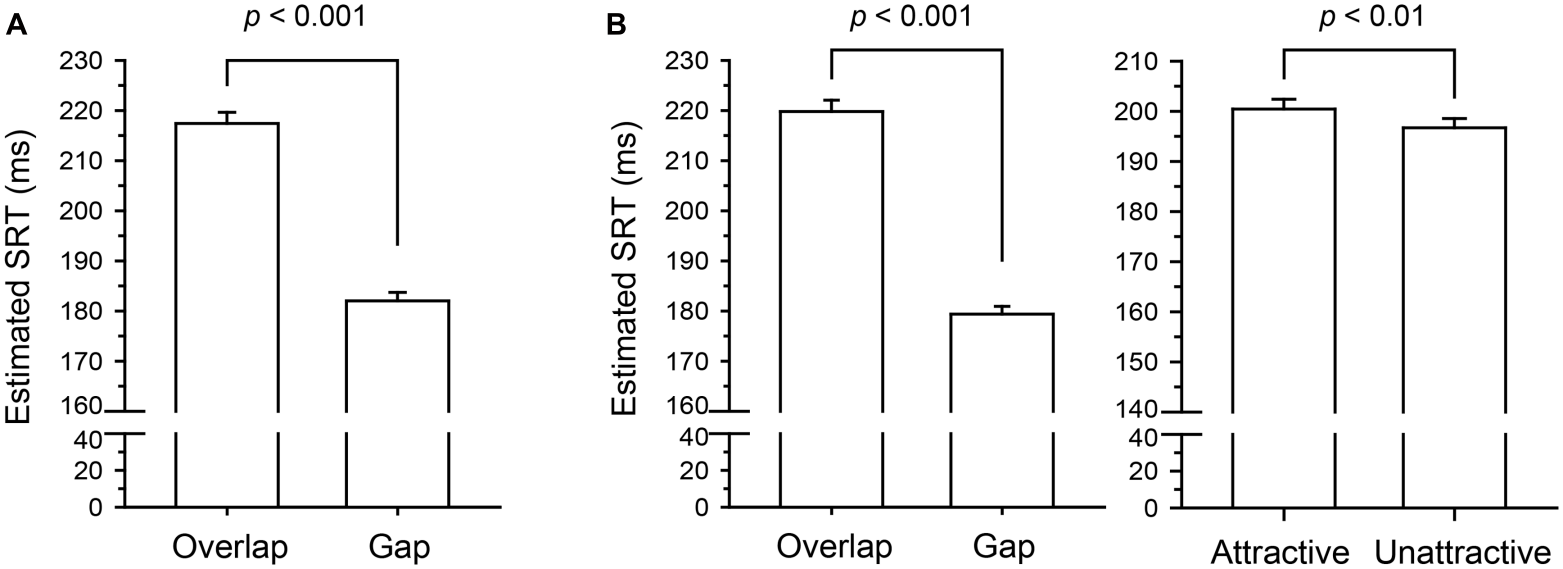
**Saccadic reaction times (SRTs) per factor level as estimated by the final linear mixed effects models (LMMs) in Experiment 1.** For better interpretability, the estimated logSRT for each factor level was transformed back to the original millisecond scale for plotting. Error bars represent +1.96 *SEM* after removing random effect variances. **(A)** Model results for the morphed faces. The data yielded a significant effect of the gap manipulation with lower SRTs in the Gap than in the Overlap condition. **(B)** Model results for the natural faces. The gap effect was qualitatively identical to the results from the morphed faces. In addition, the data yielded an effect of facial attractiveness, with longer SRTs with attractive than with unattractive faces.

***Model results: slower disengagement from attractive faces.*** Estimated SRTs per condition are depicted in **Figure 4**. The model for the morphed faces (intercept *B* = 5.29, *SE* = 1.78 × 10^-2^) yielded a significant gap effect with logSRTs being significantly shorter in gap trials as compared to overlap trials, *B* = -1.78 × 10^-1^ (*SE* = 1.48 × 10^-2^), *t* = –12.0, *p* < 0.001. Similarly, the model for natural faces (intercept *B* = 5.29, *SE* = 1.89 × 10^-2^) resulted in the expected gap effect, with logSRTs being shorter in gap trials as compared to overlap trials, *B* = –2.03 × 10^-1^ (*SE* = 1.53 × 10^-2^), *t* = –13.29, *p* < 0.001. Importantly, this model also yielded a significant effect of facial attractiveness: logSRTs were significantly shorter with unattractive compared to attractive faces, *B* = –1.87 × 10^-2^ (*SE* = 6.94 × 10^-3^), *t* = –2.69, *p* = 0.007. The interaction between the fixed effects of gap and facial attractiveness was not statistically significant, *t* = 0.35, *p* = 0.726.

### DISCUSSION

Experiment 1 tested whether (a) attentional disengagement from attractive faces is slower than from less attractive faces, (b) women and men differ in how they attend to opposite-gender versus same-gender faces, and (c) there are specific eye color preferences in opposite-gender faces in men (Laeng et al., 2007; Bovet et al., 2012). Our eye tracking experiment resulted in evidence for hypothesis (a) but did not support claims (b) and (c), i.e., we found no evidence that interactions between participants and face traits co-determine attentional disengagement from faces. In addition to the eye tracking experiment we also collected subjective attractiveness ratings for natural and morphed faces. Only for the highly attractive morphed faces, the data yielded evidence for a gender-based interaction: men as well as women gave higher ratings to feminine faces than to masculine faces but this bias was much larger in men than in women. This begs the question of why we were unable to detect the gender-based interaction at least in the morphed faces with our eye tracking experiment. In principle, it is possible that specific individual preferences for attractive facial features affect attentional processes but not necessarily the process of disengagement. Hence, in Experiment 2 we created an *attentional capture test*, asking participants to make a saccade *toward* one of two faces.

## EXPERIMENT 2: ATTENTIONAL CAPTURE BY FACES

Instead of presenting only one face at screen center, we presented two faces at different locations in the periphery and asked participants to make a saccade toward one of these locations (and ignore the other). When multiple stimuli are in the visual field they compete for attention and cognitive processing by activating their respective neural representations (Desimone and Duncan, 1995). This competition for attentional priority is determined by stimulus-driven influences, such as the strength of the visual signal and by observer-based top–down influences (e.g., expectations, goals, or memory). A suitable task to measure these early processes of attentional capture is the so called ‘*dot probe’* or *‘double cueing’* task (MacLeod et al., 1986) which has been often used in experimental psychopathology studies (e.g., Frewen et al., 2008; Yiend, 2010). The basic procedure implies a brief presentation of two task-irrelevant images (the cues) at a certain eccentricity left and right of screen center. Directly afterward, an unrelated target stimulus – often called the ‘dot probe’ – is presented either at the left or at the right position and subjects are asked to (usually) manually report the presence, identity, or position of the dot. If the dot probe appears at an attended location, RTs to the dot should be significantly faster than if the dot appears at an unattended location. This procedure has been adapted in various ways to study different questions about individually varying attention.

Here, we adapted a version of the dot-probe task which was used to study attentional biases in eating disorders (Blechert et al., 2010). We used two photographs of faces as cues. Instead of extinguishing both photos and replacing one of them with a probe that was unrelated to the images, we kept the faces visible and presented two differently colored frames around them. The participants were instructed to make a saccade to one pre-defined target color frame and ignore the differently colored distractor frame. Keeping the photos on screen and requiring participants to make a saccade toward one of the cued locations allows insight into the process of attentional capture. More specifically, it allows inferring which of the two concurrently presented faces captures attention more readily. Compared to the classical version of the dot probe task which requires manual button presses, saccades are a more ecological response when studying attentional capture (Kowler et al., 1995; Deubel and Schneider, 1996). Additionally, this procedure enables us to gage the temporal properties of attentional capture by varying the stimulus onset asynchrony (SOA) which is the interval between the appearance of the face cues and the target/distractor rectangles. We used two short intervals (150, 250 ms) and one long interval (1 s). These values are based on the literature on the so-called *‘inhibition of return’* (IOR) effect, which describes the often observed finding that attention is first captured by a particular stimulus location but if no target is presented soon afterward, this particular location is actively inhibited. This results in prolonged RTs when responding to a target presented at a cued location after about 300 ms (Posner and Cohen, 1984; Taylor and Klein, 1998; Klein, 2000). Using this procedure, we studied whether interactions between participant’s sex and eye color and the respective facial characteristics co-determine the capture of attention by attractive faces.

### METHODS

#### Participants

Forty new participants with a mean age of 22 years (*SD* = 2.7) were recruited from the same student population as in Experiment 1 to four groups of ten, resulting from crossing the variables participant sex (female vs. male) and eye color (blue vs. brown).

#### Apparatus

Setup and recording were identical to Experiment 1, with the exception that a drift check was conducted ahead of every trial.

#### Stimuli and procedure

**FIGURE 5:**
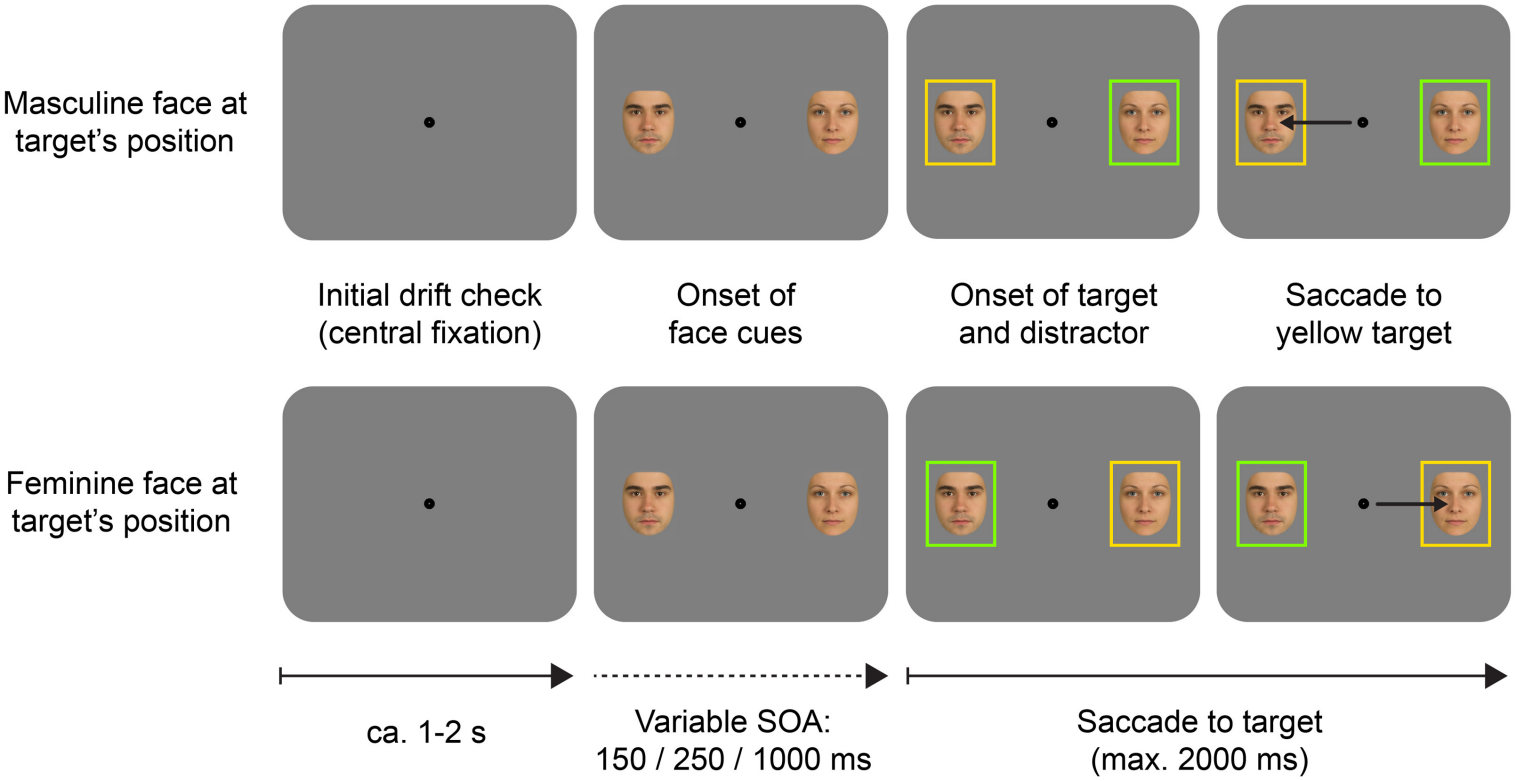
**Example schematic trials in the cueing task of Experiment 2.** In this example, the target color is yellow, and the distractor color is green (this was counterbalanced across participants). Phenotypic features of the face cues were non-predictive of the upcoming target’s location. SRT to the target was analyzed as a function of the face cue presented at the target, and the distractor location, as well as the stimulus onset asynchrony (SOA). Arrows in the rightmost pictures indicate saccades. Arrows below the pictures indicate the flow of time.

In Experiment 2 only the morphed faces served as stimuli (see **Figure 1**). We used eight different feminine and eight different masculine faces. Each face was presented with two different eye colors (blue and brown) to the same participants, resulting in 16 feminine and 16 masculine face images altogether. At the start of each session, participants were informed that the purpose of the experiment was to study the effect of human faces on visual attention and were given basic task instructions (e.g., ‘fixate on the screen center until two colored boxes appear; as soon as you see the boxes, look at the yellow box as quickly as possible’). The experiment comprised 576 trials which were randomly assigned to six blocks of 96 trials (between blocks, participants were allowed to rest briefly). In 384 trials the two simultaneous face cues showed the exact opposite phenotypic traits (e.g., the face at the target’s location was feminine with blue eyes, and the face at the distractor location was masculine with brown eyes). In the remaining 192 trials, the two faces shared either their eye color (e.g., blue-eyed feminine face and blue-eyed masculine face) or gender (e.g., blue-eyed feminine face and brown-eyed feminine face). Face identity, gender, and eye color were uncorrelated with target and distractor positions, hence face features where uninformative about target location. **Figure 5** illustrates two example trials.

Every trial started with the presentation of a central fixation target for a drift check. Then, two faces were presented to the left and to the right of the central fixation. After a variable SOA (150 ms/250 ms/1 s) two colored rectangles appeared concomitantly to frame the faces. The task of the participants was to make a saccade to the target rectangle, which was defined by color. The target color could be either green (CIE L* = 94.5, a* = –77.3, b* = 79.4) or yellow (CIE L* = 94.6, a* = –4.7, b* = 85.3), while the other color was used for the distractor. The target and distractor colors were counterbalanced across participants, announced in the initial instructions, and retained throughout the experiment. At the start of each session, participants practiced some trials to become familiar with the task and the stimuli.

Because of lower acuity in the visual periphery, the faces had to be displayed at a larger size than in Experiment 1. The appropriate size was determined by pre-tests using various stimulus sizes until a quick and reliable discrimination of gender and eye color at peripheral locations was secured. Hence, faces were shown at a size of 4 × 5° and the target and distractor rectangles had a size of 5.6 × 6.8° with line strengths of 0.25°. Faces and rectangles were presented at an eccentricity of 7.5° from the central fixation. Following the experimental blocks, participants subjectively rated the attractiveness of each face in a separate rating block where the procedure was identical to Experiment 1. In total, the data collection lasted about 80 min per participant (including setup, experiment, rating, and participant debriefing).

#### Data analysis

Data processing was done using the same software packages and settings as in Experiment 1. In total, we recorded SRTs from 23,040 trials (576 trials from each of the 40 participants). Out of the complete dataset, 876 trials (3.8%) were excluded due to the same criteria as in Experiment 1 in addition to trials in which the first saccade was erroneously directed to the distractor instead of the target rectangle.

### RESULTS

#### Subjective attractiveness ratings

***Model selection: evidence for gender interaction in morphed faces.*** Results were similar to Experiment 1 and are presented in **Table 4**. According to the (decreasing) AIC and the significant likelihood-ratio test, an appropriate model for our rating data was mor_1_, a model that included the interaction of participant sex and face gender (GI). In contrast, including the full interaction of participant sex and eye color and face gender and eye color (EGI) was not corroborated by the data.

**Table 4:** Comparison of nested LMMs fitted to ratings of morphed faces in Experiment 2.

			Model comparison			
Model	*df*	Formula	AIC	χ^2^	*df*	*p*
mor_0_	4	Attractiveness ∼ 1 + RE	5438			
mor_1_	7	Attractiveness ∼ 1 + GI + RE	5433	11.16	3	0.011
mor_2_	19	Attractiveness ∼ 1 + EGI + RE	5452	5.23	12	0.950

*Attractiveness, individual subjective attractiveness judgments; formula, model definition for the *lme4* software package; RE, random effects structure [(1| Participant) + (1| Stimulus)]. GI = [Participant Sex × Face Gender]. EGI = [Participant Sex × Participant Eye Color × Face Gender × Face Eye Color].*

***Model results: preference for highly attractive feminine faces in men.*** Testing the fixed effects part of the final model mor_1_ confirmed the significant interaction of participant sex and face gender, *F*(3,97.1) = 3.73, *p* < 0.014. **Table 5** shows the fixed effect estimates reflecting the rating bias toward feminine faces for men and women separately. The tendency for rating feminine faces as more attractive than masculine faces was present in female as well as male participants, but this bias was only significant in male participants.

**Table 5:** Fixed effect estimates for a rating bias toward feminine morphed faces in Experiment 2.

Fixed effect	*B (SE)*	*t*	*p*
(Intercept)	4.10 (0.125)		
Female participants (feminine – masculine)	0.31 (0.178)	1.74	0.088
Male participants (feminine – masculine)	0.53 (0.178)	2.99	0.004

*Results based on individual ratings on a 7-point scale. Masculine, ratings of masculine morphed faces; feminine, ratings of feminine morphed faces. Positive *B* and *t* values indicate that feminine faces received higher ratings than masculine faces.*

#### Saccadic reaction times

***Model selection: evidence for gender interaction in attentional capture.*** To determine whether participants’ attention was biased toward a particular face gender and eye color depending on their own expression of these traits, we analyzed the obtained SRTs separately with respect to (a) the target face cue properties, and (b) the distractor face cue properties. In all analyses of SRTs, we also modeled the effect of SOA, as we hypothesized that any individual preferences might show a different pattern with the shorter as compared to longer SOAs. In all models, we included random intercepts for combinations of particular target and distractor faces. **Table 6** presents the results.

**Table 6:** Comparison of nested LMMs fitted to log-transformed SRTs in Experiment 2.

			Model comparison			
Model	*df*	Formula	AIC	χ^2^	*df*	*p*
**Target face cues**
targ_0_	6	logSRT ∼ 1 + SOA + RE	–1576			
targ_1_	15	logSRT ∼ 1 + SOA × TGI + RE	–1577	18.88	9	0.026
targ_2_	51	logSRT ∼ 1 + SOA × TEGI + RE	–1544	38.21	36	0.369
**Distractor face cues**
dist_ 0_	6	logSRT ∼ 1 + SOA + RE	–1576			
dist_1_	15	logSRT ∼ 1 + SOA × DGI + RE	–1583	24.63	9	0.003
dist_2_	51	logSRT ∼ 1 + SOA × DEGI + RE	–1540	28.79	36	0.798

*logSRT, individual log-transformed SRTs; formula, model definition for the *lme4* software package; SOA, stimulus onset asynchrony; RE, random effects structure [(1| Participant) + (1| Stimulus)]. TGI = [Participant Sex × Target Face Cue Gender]. TEGI = [Participant Sex × Participant Eye Color × Target Face Cue Gender × Target Face Cue Eye Color]. DGI = [Participant Sex × Distractor Face Cue Gender]. DEGI = [Participant Sex × Participant Eye Color × Distractor Face Cue Gender × Distractor Face Cue Eye Color].*

The model comparison showed that including the interaction between participant sex and target’s face cue gender (TGI), or the interaction between participant sex and distractor’s face cue gender (DGI) improved goodness of fit over the respective baseline models. However, the present data yielded no evidence for EGIs (TEGI/DEGI) in any of the tested models. Hence, our data suggest that male and female participants differed in how quickly they made saccades to the target rectangle depending on whether the face shown at the target location was masculine or feminine (and whether the face at the distractor location was masculine or feminine, respectively). To further scrutinize this interaction we looked at the estimates of these models in more detail.

**FIGURE 6:**
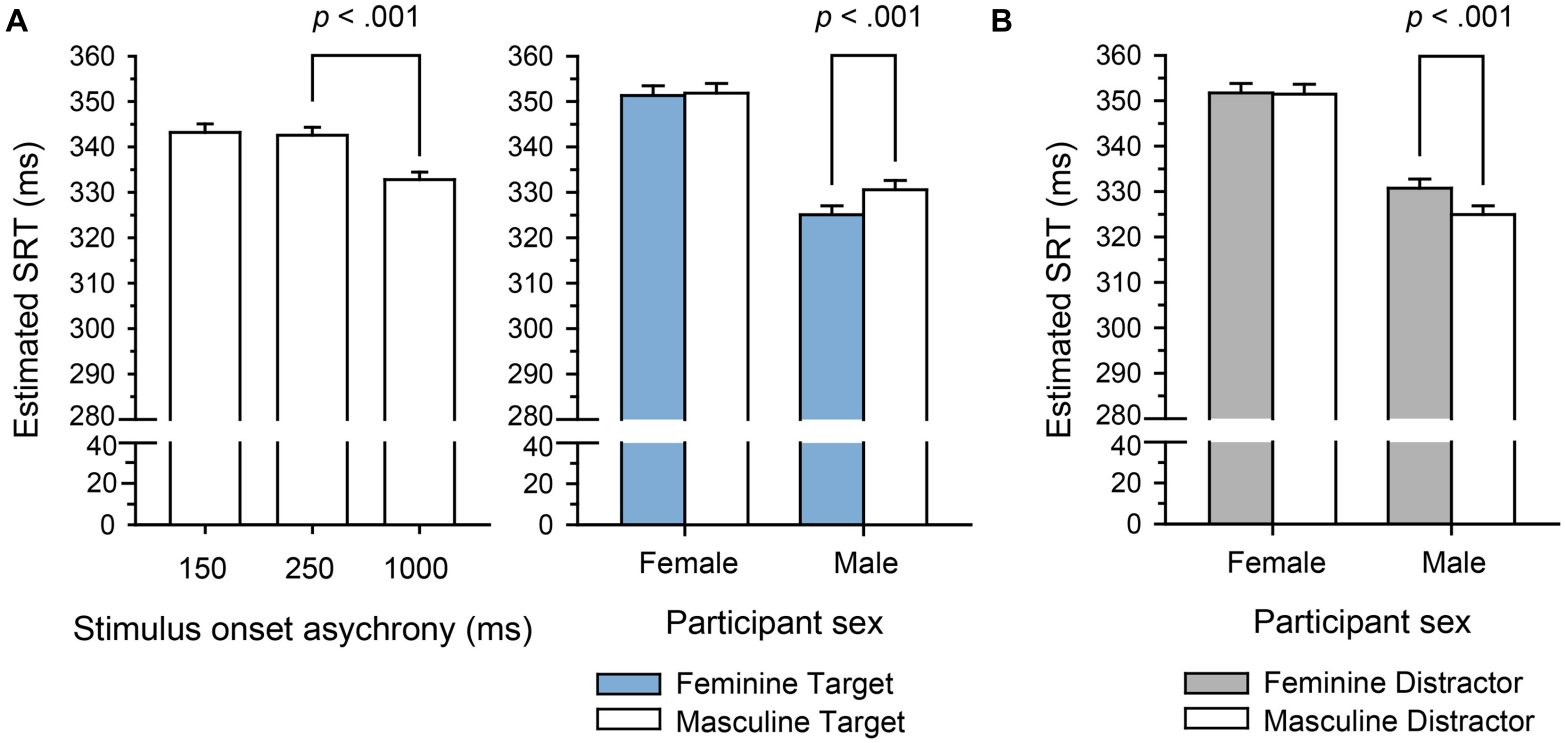
**Saccadic reaction times per factor level as estimated by the final LMMs in Experiment 2.** For better interpretability, the estimated logSRT for each factor level was transformed back to the original millisecond scale for plotting. Error bars represent +1.96 *SEM* after removing random effect variances. **(A)** Model results for the target face cues. The data yielded a significant effect of the SOA manipulation and a significant interaction of participant sex and target face cue gender. For male participants, SRTs were shorter when the face at the target location was feminine. **(B)** Model results for the distractor face cues. The model yielded a significant interaction of participant sex and distractor face cue gender. For male participants, SRTs were longer when there was a feminine face at the distractor location (the SOA effect is not depicted for the distractor model as it was qualitatively identical to the target model).

***Model results: men’s attention is more effectively captured by feminine faces.*** Estimated SRTs are depicted in **Figure 6**. Testing the fixed effects in the final target face cue model (targ_1_) confirmed our conclusions from the model comparisons. The SOA effect, *F*(2,40) = 40.17, *p* < 0.001, as well as the TGI interaction effect, *F*(3,104) = 4.77, *p* = 0.004, were significant. The interaction between these two was not significant, *F*(6,178) = 0.77, *p* = 0.594. Results for the corresponding distractor face cue model (dist_1_) followed the same pattern: the overall SOA effect, *F*(2,40) = 40.25, *p* < 0.001, and the overall DGI effect, *F*(3,104) = 5.28, *p* = 0.002, were significant. Again, the interaction between them was not significant, *F*(6,165) = 1.47, *p* = 0.184. To scrutinize these effects (in particular the gender based differences) we looked at the models’ contrast estimates which are listed in **Table 7**.

**Table 7:** Fixed effect estimates for SOA and GI parameters in the final LMMs of log-transformed SRTs in Experiment 2.

Fixed effect	*B (SE)*	*t*	*p*
**Target model**
(Intercept)	5.83 (0.034)		
SOA_250_–SOA_150_	–1.8 × 10^-3^ (3.83 × 10^-3^)	–0.46	0.642
SOA_1000_–SOA_250_	–2.9 × 10^-2^ (3.84 × 10^-3^)	–7.50	<0.001
Female_mas_ – Female_fem_	1.5 × 10^-3^ (4.59 × 10^-3^)	0.32	0.749
Male_mas_ – Male_fem_	1.7 × 10^-2^ (4.62 × 10^-3^)	3.64	<0.001
**Distractor model**
(Intercept)	5.83 (0.034)		
SOA_250_–SOA_150_	–1.8 × 10^-3^ (3.83 × 10^-3^)	–0.47	0.641
SOA_1000_–SOA_250_	–2.9 × 10^-2^ (3.83 × 10^-3^)	–7.51	<0.001
Female_mas_ – Female_fem_	–7.5 × 10^-4^ (4.59 × 10^-3^)	–0.16	0.870
Male_mas_ – Male_fem_	–1.8 × 10^-2^ (4.62 × 10^-3^)	–3.84	<0.001

*SOA, stimulus onset asynchrony (150/250/1,000 ms); female, female participants; male, male participants; mas, masculine faces; fem, feminine faces.*

For both models, the estimated SOA effects showed that saccades were initiated significantly faster after the (long) 1 s SOA than after the (short) 250 or 150 ms SOAs, while there was no difference between the latter two. Interestingly, our results confirmed an attentional bias for feminine faces, exclusively for male participants: saccades of male participants toward the target rectangle were initiated significantly faster if a feminine face was presented at the target location, and significantly slower if a feminine face was presented at the opposite, distractor location. In contrast, SRTs of female participants were not affected by the gender of the face stimulus at either of the locations.

### DISCUSSION

Experiment 2 followed up on the aim to investigate interactions between participant traits and face stimulus characteristics (eye color and gender) in the attentional bias for attractive faces. There was one important conceptual difference to Experiment 1: we measured attentional capture *toward* one out of two simultaneously presented faces and not *disengagement* from one fixated face. Hence, Experiment 2 targeted a sub-process of attention that precedes disengagement (cf. Posner and Petersen, 1990). In addition, by presenting two faces simultaneously in the visual periphery we created the necessary preconditions for biased competition between faces – a viewing situation that more closely mirrors attentional orienting toward particular individuals in a social world. This ultimately enabled us to establish the hypothesized interaction of participant sex and face stimulus gender, reflecting an attentional bias toward attractive feminine faces in men. Again, we supplemented our eye tracking data with subjective attractiveness judgments. The latter revealed that men judged feminine faces as more attractive than masculine faces while this bias toward feminine faces was much weaker (and non-significant) in women.

In addition, we manipulated the interval between the onset of the face cues and the onset of the targets in order to measure the temporal properties of attentional deployment to the face cues. We expected an IOR effect as usually observed with SOAs exceeding 300 ms (e.g., Klein, 2000): an interaction between cueing (by preferred faces) and the length of the interval reflecting that attention is initially captured by an attractive face and later inhibited at the same location. This should have resulted in prolonged RTs to the location where attention was captured by an attractive face with the 1 s SOA. Also, because only men showed a preference for feminine faces, the IOR effect was expected to be present in men only. This IOR should then be reflected in an interaction between the GI and SOA’s fixed effects. However, this interaction was not observed. Rather, we found a general acceleration of SRTs after the longest SOA. One possible explanation for this finding could be that the longest SOA implicated a better temporal warning signal of the target due to the large temporal gap between the middle (250 ms) and long SOA (1 s) which could modulate SRTs independently and in addition to any face-based spatial attention effects (e.g., Walker et al., 1995).

Our main findings are in line with previous literature showing that men exhibit stronger preferences for attractive opposite-sex faces than women. Yet, even with our modified experimental procedure our data did not support the hypothesis that eye color additionally interacts with individual preferences – as predicted by Laeng et al. (2007) who found that blue-eyed men rated blue-eyed women as more attractive than brown-eyed women and who explained this with an evolutionary adaptive strategy of blue-eyed men to maximize their subjective assurance of paternity. It is worth pointing out that the present study had a smaller number of participants (per eye color and sex group, respectively) than the original Laeng et al. (2007) study. In principle, one might suspect that the original finding was not corroborated because of low statistical power. However, taking the originally reported effect size into account, it is unlikely that our study would fail to capture such a large effect in all datasets that we collected throughout this study, if it is present.

## GENERAL DISCUSSION

The current study tested potential contributions of gender and eye color to individual differences in preferences for attractive faces and the respective effects on two distinct attentional processes: disengagement of attention (Experiment 1) and capture of attention (Experiment 2). In prior studies of looking times at attractive faces, these phases were generally confounded and it could not be decided which sub-process was responsible for the effects. For our tests, we chose SRTs rather than looking times, because saccades are tightly coupled to the engagement of attention (Kowler et al., 1995; Deubel and Schneider, 1996). Also we conducted our tests with a focus on two sources of inter-individual differences in attention to faces. First, we studied if men and women showed distinct patterns of attentional processes in response to same-sex and opposite-sex faces, respectively. This was confirmed for attentional capture (in Experiment 2) but not for the disengagement of attention (in Experiment 1). Second, we studied whether eye color could explain additional differences between participants, such as an attentional bias of blue-eyed men toward blue-eyed feminine faces. The latter could not be confirmed in any of our experiments. Thus, our results show that individual preferences for attractive faces are partly reflected in respective differences in visual attention but not all of these inter-individual differences are equally robust.

This brings us to an important related point. Our results differ from previous reports because we were unable to replicate the finding that blue-eyed men consider blue-eyed women more attractive than brown-eyed women (Laeng et al., 2007). One reason for the failure to replicate the original finding could be that the stimuli of Laeng et al. (2007) were more naturalistic portrait photographs including features beyond the face itself (such as hairstyles and clothing). For the present study, we constructed new stimuli and minimized any potentially confounded features that could attract attention independently of face gender and eye color. However, it is possible that contextual features are necessary to induce stronger individual evaluative and behavioral preferences so that the use of the stronger constrained stimuli in the present experiments prevented us from replicating the original finding. Another possibility is that the eye color may have been more salient and easier to discriminate in the original study of Laeng et al. (2007) where faces were shown full-screen on an 11.4′′ monitor whereas their size was smaller in the present study (Experiment 1: 2.7 × 3.2°; Experiment 2: 4 × 5°).

What is even more important in our view, the result of a study also hinges on the specific statistical procedures applied to the collected data. Inconsistencies across studies could stem from distorted data due to averaging across subjects, stimuli, or conditions without accounting for random variance that is not generalizable to the independent variables of the design (e.g., Wells and Windschitl, 1999; Judd et al., 2012). This is particularly problematic for studies addressing interactions between groups of participants and experimental stimuli. While this problem was taken care of with the present LMM analyses, spurious interactions might become significant with more traditional statistics. Also, the exclusive use of the classic approach of null hypothesis significance testing (NHST) has been often criticized (e.g., Bakan, 1966; Greenwald, 1975; Cohen, 1994; Loftus, 1996; Sohn, 1998; Nickerson, 2000) and more informative statistical tools, such as model comparisons and measures of Information Theory and Bayesian statistics have been advocated recently (e.g., Glover and Dixon, 2004; Stephens et al., 2005; Wagenmakers, 2007).

## CONCLUSION

In the present paper we linked research on individual preferences for attractive faces to inter-individual differences in visual attention toward faces of varying attractiveness. Using a combination of well-controlled experimental approaches and linear mixed effects modeling, we replicated previous results showing that attractive faces lead to longer dwell times. In addition, we found evidence for gender-based differences in attentional capture. We could not replicate a previously reported EGIs and close with a recommendation for the statistical analysis of inter-individual differences in general.

## Conflict of Interest Statement

The editor Ulrich S. Tran declares that, despite being affiliated at the same institution as the authors Christian Valuch, Lena S. Pflüger, Bernard Wallner and Ulrich Ansorge, the review process was handled objectively and no conflict of interest exists. The authors declare that the research was conducted in the absence of any commercial or financial relationships that could be construed as a potential conflict of interest.
